# First parechovirus reported case in Saudi Arabia in hospitalized immunocompromised adult patient

**DOI:** 10.1186/s12985-024-02372-4

**Published:** 2024-05-02

**Authors:** Abdullah Alshammari, Jawaher Alotaibi, Reem Almaghrabi, Reema Bawazeer, Sahar Althawadi, Hamsa Tayeb

**Affiliations:** 1 Department of Medicine, King Faisal Specialist Hospital and Research Center, Riyadh, Kingdom of Saudi Arabia; 2 Organ Transplant Center of Excellence, King Faisal Specialist Hospital and Research Center, Riyadh, Kingdom of Saudi Arabia; 3 Center of Genomic Medicine CGM, King Faisal Specialist Hospital and Research Center, Riyadh, Kingdom of Saudi Arabia; 4 Microbiology Laboratory, Department of Pathology & Laboratory Medicine, King Faisal Specialist Hospital and Research Center, Riyadh, Kingdom of Saudi Arabia; 5 Clinical Scientist, Head of Functional Genomic section, Transitional Genomic (TG) Department, Center of Genomic Medicine CGM, King Faisal Specialist Hospital and Research Center, P.O.Box 3354, MBC-03-06, Riyadh, 11211 Kingdom of Saudi Arabia

**Keywords:** Human parechovirus, Encephalitis, Shotgun whole genomic sequencing, And annotation

## Abstract

Human parechovirus, a member of the *Picornaviridae* family (PeVs), can lead to severe infections, including severe meningitis, meningoencephalitis, and sepsis-like syndrome. We report a case of human parechovirus-related encephalitis in a 52-year-old woman diagnosed with glioblastoma multiforme. She underwent surgical resection in June 2022. Unfortunately, her disease recurred, and she underwent a second resection in August 2022, followed by radiation therapy and Temozolomide therapy. She presented to the hospital with acute confusion followed by seizures, necessitating intubation for airway support. A cerebrospinal fluid (CSF) sample was obtained and processed using the Biofire FilmArray, which reported the detection of HSV-1. Despite being on Acyclovir, the patient did not show signs of improvement. Consequently, a second CSF sample was obtained and sent for next-generation sequencing (NGS), which returned a positive result for Parechovirus. In this presented case, the patient exhibited symptoms of an unknown infectious cause. The utilization of NGS and metagenomic analysis helped identify Parechovirus as the primary pathogen present, in addition to previously identified HSV. This comprehensive approach facilitated a thorough assessment of the underlying infection and guided targeted treatment. In conclusion, the application of NGS techniques and metagenomic analysis proved instrumental in identifying the root cause of the infection.

## Introduction

Human parechovirus (PeVs) belongs to the *Picornaviridae* family. *Picornaviridae* are non-enveloped, positive-sense, single-stranded RNA viruses [1]. PeV infections have been predominantly recognized in the pediatric population [2]. Clinical presentations of PeV infections can vary from asymptomatic to more severe forms of the disease. PeVs share the same taxonomic family as enteroviruses. There are four types of PeV. Type A is typically associated with mild infections, while type A3 has been linked to severe infections, including severe meningitis, meningoencephalitis, and sepsis-like syndrome [1, 2].

In recent years, the adoption of whole-genome sequencing (WGS) for pathogen identification has garnered increasing attention within the scientific community. This heightened interest can be attributed to the limitations observed in traditional molecular and serological methods, which have shown deficiencies in detecting pathogens responsible for infectious diseases. The potential of WGS lies in its ability to improve diagnostic speed and accuracy, ultimately facilitating more effective therapeutic interventions. Given these potential advantages, it is imperative to acknowledge that incorporating WGS into routine pathogen identification protocols can provide early diagnosis and prompt initiation of appropriate treatment [3–6].

## Case report

A 53-year-old female recently received a diagnosis of Glioblastoma Multiform and underwent surgical resection twice, first in June 2022 and then again in August 2022, due to disease relapse. Her treatment plan included sessions of radiation therapy, Dexamethasone, and Temozolomide. Her post-operative recovery was uneventful, but her family brought her to the emergency department 1 month after her second surgery due to fever, acute confusion, blurry vision, right-sided weakness, and an inability to walk. During her hospitalization, her condition deteriorated further. She experienced seizures that necessitated sedation and intubation, and subsequently, she was initiated on high-dose antimicrobials due to suspicion of post-neurosurgical meningitis.

**Fig. 1:**
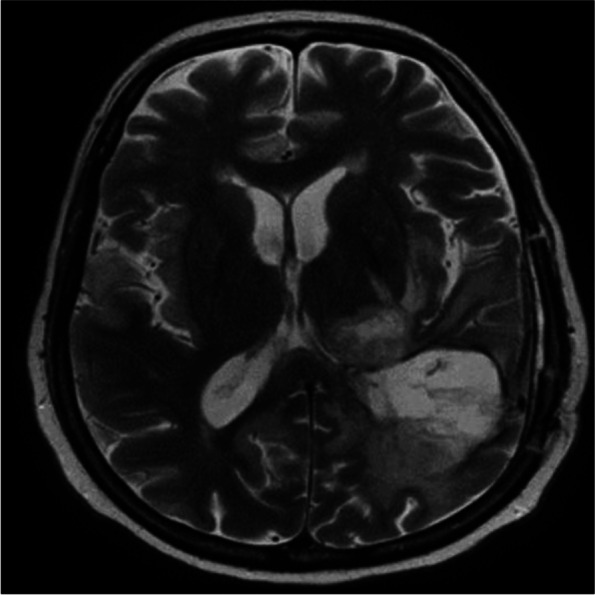
Upon analyzing the patient's brain MRI, increased hyperintensity and diffusion restriction signals were observed in several areas, including the right temporal lobe, right mesial temporal structure, insular cortex, inferior frontal lobe, and cingulate gyrus, leading to suspicions of viral encephalitis

Upon admission, her initial investigations were unremarkable except for hyponatremia, with sodium levels measuring 126 mmol. Additionally, a CT scan of her brain revealed post-surgical changes without any other concerning abnormalities. In response to her seizure episode, an electroencephalogram was performed, which identified moderate encephalopathy and disturbances in cerebral activity in the right temporoparietal region, accompanied by active epileptic discharges. Despite medical intervention, the patient’s condition persisted, and a follow-up electroencephalogram showed refractory seizure activity, leading to several antiepileptic medications. Subsequent magnetic resonance imaging (MRI) of her brain indicated increased hyperintensity and diffusion restriction signals in the right temporal lobe, right mesial temporal structure, insular cortex, inferior frontal lobe, and cingulate gyrus, raising suspicions of viral encephalitis (Fig. 1). The results of the cerebrospinal fluid analysis after 3 days of antimicrobial treatment for meningitis are shown in Table 1.

**Table 1:** Shows the results of the cerebrospinal fluid analysis after 3 days of antimicrobial treatment for meningitis

RBC 116 cells	Normal range 0–5 cells
WBC 16 cells	Normal range < 5 cells
Lymphocyte 25%	
Glucose 4.47 mmol/L	Normal range 2.2–3.9 mmol/L
Protein 652 mg/dl	15–60 mg/dl

**Fig. 2:**
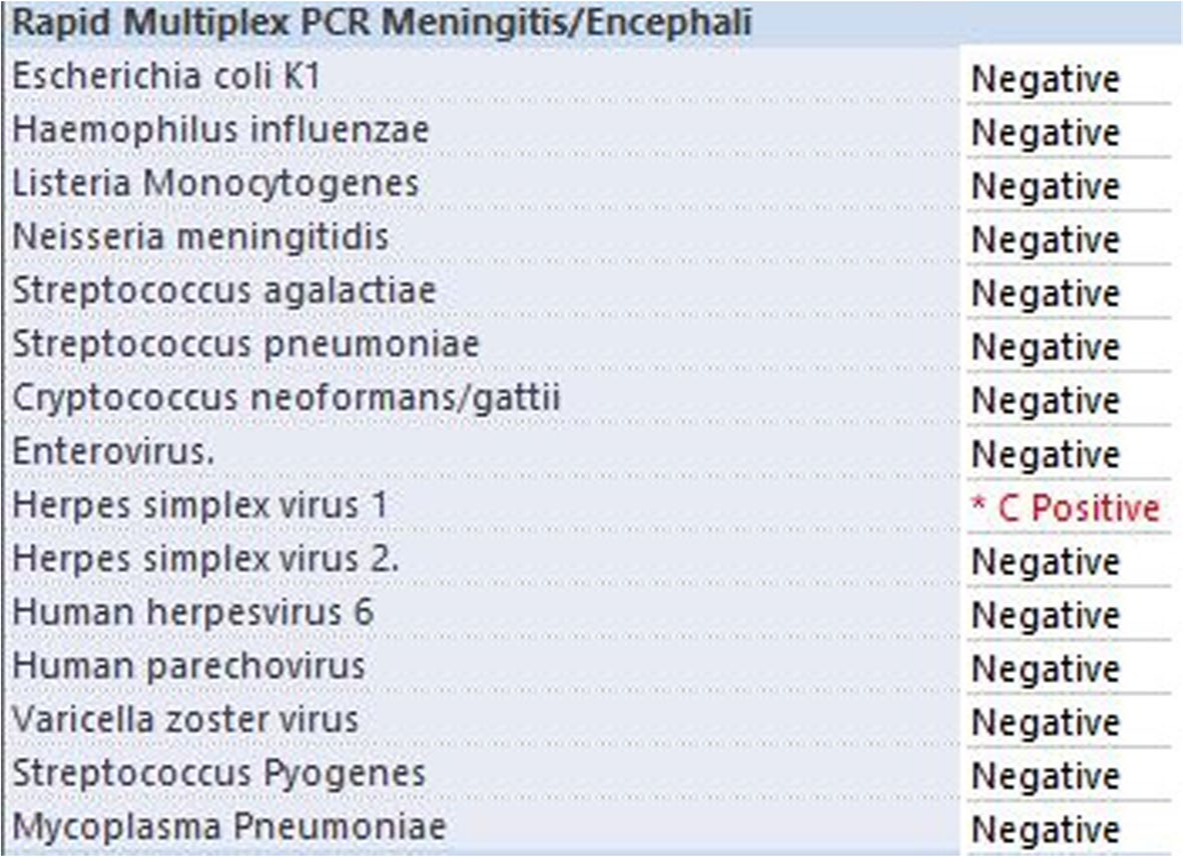
Microbiological results of the CSF sample, including common bacterial and viral pathogens causing meningitis, were positive for Herpes simplex 1

Microbiological studies on this CSF, including common bacterial and viral pathogens causing meningitis, were positive for Herpes simplex 1 PCR (Fig. 2).

**Fig. 3:**
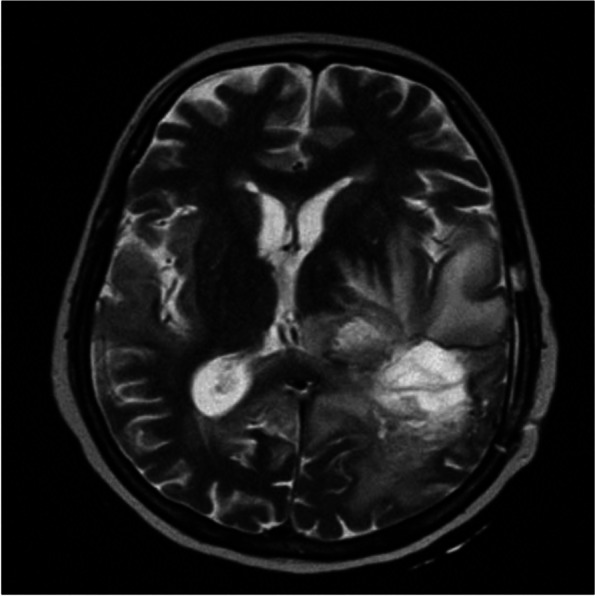
This MRI figure shows that the distribution of parenchymal injury is consistent with herpetic encephalitis, along with the progression of high-grade glioma and persistent entrapment of the left temporal horn

Acyclovir at a dosage of 10 mg per kg every 8 hours was added to her antimicrobial regimen. The patient’s level of consciousness remains depressed, and she continues to run a low-grade fever despite receiving antiviral and antibacterial therapy. There has been no improvement in her level of consciousness during this period. A follow-up magnetic resonance imaging (MRI) was conducted after 10 days of acyclovir treatment; unfortunately, her consciousness level did not improve. The MRI report indicated the progression of right hemispheric encephalitis with features suggestive of associated meningitis. The distribution of parenchymal injury is consistent with herpetic encephalitis, along with the progression of high-grade glioma and persistent entrapment of the left temporal horn (Fig. 3).

As a result of the MRI findings, the case was reassessed by a multidisciplinary team, which included experts from neurology, neurosurgery, infectious diseases, and oncology. The consensus was to perform a repeat lumbar puncture to assess for any neurovascular complications related to HSV encephalitis, especially since the patient’s level of consciousness did not improve. The repeated cerebrospinal fluid (CSF) analysis showed no pleocytosis, and the repeated Herpes simplex PCR was negative. Metagenomics testing was conducted to assess for possible mutant HSV, considering the reported progression on her follow-up MRI. Her antiviral agents were modified to Foscarnet while awaiting the results of the metagenomics test. She completed more than 14 days of Foscarnet treatment and 21 days of acyclovir.

Metagenomics analysis revealed the presence of Para-echovirus (with a coverage of 10.6%), along with other infective agents, including *Raistonia pickettii*, *Burkholderia cepcia complex*, *Elizabethkingia anopheles*, and *Pseudomonas sstatzeri* (Table 1). The patient was already receiving antibacterial treatment that covered all these pathogens, but unfortunately, her level of consciousness did not improve. Subsequent follow-up MRI scans showed a worsening of FLAIR imaging hyperintensity in the right temporal, parietal, and frontal lobes, suggesting herpetic encephalitis. Signs of laminar necrosis along the cortex of the temporal and parietal lobes were also observed, along with an increase in the dimensions of the recurrent necrotic mass (GBM) and worsening surrounding vasogenic edema and mass effect. These findings indicate an advanced stage of HSV encephalitis and a recurrence of her underlying malignancy. Despite ongoing medical care, the patient’s clinical status did not improve, and her treating oncologist eventually gave her a poor prognosis. Consequently, she was transferred back to her home country.

**Table 2:** Metagenomics analysis shows the presence of Para-echovirus, along with other infective agents

**Bacteria**	**Proportion of detected bateria**
*Raistonia Pickettii*	46.3%
*Burkholderia cepcia complex*	22.7%
*Elzabethkingia anopheles*	16.6%
*Psuedomonas sstatzeri*	14.4%
**Viruses**	**Proportion of detected Viruses**
*Human parecchvirus*	100%

### Literature review

Parechovirus can cause infections in humans, particularly in infants and young children. It was initially identified in the 1950s, but it was not until the 1990s that it gained recognition as a significant cause of neonatal sepsis and central nervous system (CNS) infections. Subsequent studies have demonstrated that parechoviruses can lead to a wide range of clinical conditions, spanning from mild respiratory infections to severe CNS infections such as meningitis or encephalitis. While parechovirus infections are generally self-limiting and mild, severe cases can result in long-term complications or even death. Consequently, understanding the patterns of parechovirus transmission and the clinical features of infection is crucial for effective diagnosis and treatment [7–10]. Diagnosis of PeV in pediatrics typically achieved with nucleic acid amplification testing in CSF and stool samples. Specific platforms for rapid molecular diagnostics include PeV in meningitis multiplex polymerase chain reactions, such as the total nucleic acid isolation kit (Roche Diagnostics, Mannheim, Germany) [1].

One study examined the prevalence of Parechovirus in adults in the Netherlands. Out of more than 10,000 clinical samples, approximately 11 samples tested positive for PeV by PCR. Among these patients, 8 were found to be immunocompromised and developed disseminated disease with infections lasting for more than 3 months. This indicates that immunocompromised individuals, whether due to an underlying medical condition or immunosuppressive therapy, are at a higher risk of developing a more severe form of Parechovirus infection and experiencing prolonged shedding of the virus [7].

In 2018, a noteworthy case of Human Parechovirus (PeVs) encephalitis in adults was reported, featuring a complex presentation of refractory status epilepticus [11]. As far as our current knowledge goes, there have been no documented cases of Human Parechovirus (PeVs) encephalitis among adults in Saudi Arabia. The treatment for Parechovirus in pediatric and adult patients is primarily supportive management, as there are no available antiviral therapies.

## Materials and methods

### Patient information

The patient was a 52-year-old female residing in Bahrain and from the Arab ethnicity. It is noteworthy that the patient has no history of previous medical illnesses.

### Sample collection

Regarding the sample collection, on 24 October 2023, a second lumbar puncture (LP) procedure was performed to obtain a cerebrospinal fluid (CSF) sample, which was subsequently sent for metagenomics analysis.

### DNA isolation purification and quantification

Samples sent to our laboratory were aliquoted and labeled with patient information. Nucleic acid material purification (DNA/RNA) was done in the laboratories using the “MAGMAX MICROBIOME ULTRA W/PLATE 100RXNS” kit. The isolated material was quantified using Qubit 4, and DNA purity was assessed using the NanoDrop 2000c UV-Vis spectrophotometer, with a 260/280 ratio of 1.8.

### Library preparation and sequencing

An aliquot of the extracted sample was used as input for the Illumina DNA Prep Kit for library preparation (Illumina Inc., San Diego, CA). Sample tagmentation was performed using 10 ng of template material. After tagmentation, PCR amplification was carried out according to the manufacturer’s instructions, utilizing a unique combination of indexes provided by the manufacturer to allow for sample multiplexing. Following amplification, short DNA fragments in each DNA library were removed using AMPure XP bead purification. Subsequently, the libraries were normalized to 4 nM for pooling.

To prepare for cluster generation and sequencing, equal volumes of the normalized libraries were combined, diluted in a hybridization buffer, and denatured with 0.2 M NaOH. Finally, pair-end sequencing was conducted using the MiSeq Reagent Kit v3 (600 cycles) on the Illumina MiSeq platform.

### DNA sequence analysis

For the first time in our genome laboratory, we utilized the Base-Space Sequence Hub software, which offers a wide range of next-generation sequencing (NGS) data analysis applications developed or optimized by Illumina. This software facilitated the encrypted data flow from the instrument into Base-Space Sequence Hub, allowing us to efficiently manage and analyze our data using a carefully curated set of analysis applications.

## Discussion

Human parechovirus (PeV) infections among adults are infrequent, and most documented cases manifest as either asymptomatic or mild infections. However, there have been reports of severe PeV infections linked to conditions such as encephalitis, myocarditis, muscle weakness, myalgia, orchiodynia, and fasciitis. Notably, parechovirus encephalitis in healthy populations is exceptionally rare, with the first documented adult case recorded in 2018 [8–11].

The current literature lacks reports detailing PeV infections within the Kingdom of Saudi Arabia, raising concerns about the possibility of underdiagnosis and underreporting. It is essential to consider the high prevalence of encephalitis cases with unknown sources and the fact that confirming a PeV diagnosis requires advanced diagnostic tools. These diagnostic methods are not widely available throughout the kingdom and are typically restricted to specific tertiary centers.

In the presented case, the herpes simplex virus (HSV) was initially identified in the patient’s cerebrospinal fluid (CSF), offering a plausible explanation for their symptoms. Nevertheless, after 10 days of Acyclovir treatment and subsequent magnetic resonance imaging (MRI) scans indicating continued encephalitis progression, suspicions arose regarding alternative diagnoses. Consequently, a lumbar puncture was repeated, and metagenomic analysis of the collected sample confirmed Parechovirus (PeV) infection using next-generation sequencing (NGS). This demonstrated the NGS’s ability to provide comprehensive insights.

The utilization of artificial intelligence (AI) in metagenomics analysis has brought about a revolution in the field of infectious disease diagnosis and treatment. Patients in intensive care units (ICUs) are particularly susceptible to infections and require prompt and precise diagnoses to enhance their chances of recovery. Metagenomics analysis proves invaluable for pinpointing the causative agents of infectious diseases since it permits an unbiased detection of all DNA and RNA present in a patient sample, encompassing bacteria, viruses, and fungi. AI algorithms can undergo training on extensive datasets of metagenomics data, allowing them to recognize patterns and predict potential pathogens implicated in an infection. Machine learning algorithms can enhance diagnostic accuracy and speed, facilitating timely treatment decisions. Furthermore, AI-assisted metagenomics analysis can identify potential antibiotic-resistance genes, aiding in selecting appropriate antibiotic therapy for ICU patients [12].

We aim to enhance our comprehension of PeVs epidemiology and its clinical presentations by reporting this case. Moreover, we intend to underscore the importance of maintaining a low threshold for suspicion and promptly initiating appropriate investigations into potential causes.
